# Evaluation of the Efficacy of a Vibrotactile Device for Positional Therapy of Sleep-Disordered Breathing: A Pilot Study in Healthy Volunteers

**DOI:** 10.3390/clockssleep8010014

**Published:** 2026-03-16

**Authors:** Andrey R. Alexandrov, Anton R. Kiselev, Mikhail V. Agaltsov, Anastasia R. Alexandrova, Ivan A. Kudashov

**Affiliations:** 1 Faculty of Biomedical Engineering, Bauman Moscow State Technical University, 5(1) 2nd Baumanskaya St., 105005 Moscow, Russia; 2 National Medical Research Center for Therapy and Preventive Medicine, 10(3) Petroverigsky Ln., 101000 Moscow, Russia; 3 Academy of Engineering, Patrice Lumumba Peoples’ Friendship University of Russia, 6 Miklukho-Maklaya St., 117198 Moscow, Russia

**Keywords:** obstructive sleep apnea, position-dependent sleep-disordered breathing, positional therapy, snoring, sleep quality

## Abstract

The role of body position during sleep, particularly the supine position, is now recognized as an important factor in the development of sleep-disordered breathing such as snoring, apnea, and hypopnea. This pilot study aimed to evaluate the efficacy of a new wearable vibrotactile device (SoftSleep) in reducing sleep time in the supine position without negatively affecting total sleep duration or perceived sleep quality. This pilot study included 20 healthy volunteers. Sleep was monitored over two consecutive nights: the first night without positional therapy (PT) and the second night using a PT device. The primary outcome measures were total sleep time, sleep duration in the supine position, number of position changes, and subjective sleep quality (using the modified Pittsburgh Sleep Quality Index). Use of SoftSleep showed a significant reduction in the mean proportion of sleep in the supine position from 56.01% to 7.84% (*p* < 0.001). Total sleep time did not change significantly (7:39 ± 1:33 vs. 7:42 ± 1:19; *p* > 0.05). A moderate increase in the number of position changes was not accompanied by a deterioration in subjective sleep quality: 90% of participants rated their sleep with the device as very good or fairly good. Only three participants reported brief awakenings, which did not affect their overall perception of nighttime rest. These results indicate that the SoftSleep device effectively promotes sleep in a non-supine position without altering sleep quality or subjective perception of sleep. The high tolerability of the device confirms its potential for further clinical evaluation in patients with positional sleep apnea.

## 1. Introduction

Sleep-disordered breathing (SDB) represents a continuum of respiratory pathologies that occur during sleep, ranging from simple snoring to more life-threatening conditions such as obstructive sleep apnea (OSA). Among the various SDB phenotypes, positional obstructive sleep apnea (POSA) represents a distinct and well-known clinical and polysomnographic subtype characterized by upper airway obstruction occurring predominantly or exclusively during sleep in the supine position [1].

It is well known that OSA not only negatively impacts sleep quality but is also associated with an increased risk of cardiovascular disease, excessive daytime sleepiness, and an overall decreased quality of life [2,3,4]. Several studies have also shown that certain types of SDB (such as snoring) can negatively impact the sleep quality of the bed partner. According to published data, up to 90% of bed partners of chronic snorers report insufficient rest upon awakening and decreased daytime activity [5,6].

OSA is a serious public health problem worldwide. Research suggests that approximately 1 billion people worldwide may suffer from SDB, which is nearly 10 times more than previously estimated [7]. Even more alarming findings indicate that approximately half the world’s population has signs of OSA [8].

The prevalence of OSA is believed to be increasing annually, driven by lifestyle changes, rising obesity rates, and an aging population. The main challenge in accurately estimating the number of people suffering from SDB is the low rate of diagnosis in the medical community, as well as the general underestimation of the severity of this condition among patients [9,10]. According to widely cited epidemiological data, sleep apnea affects approximately 13–33% of men [11] and nearly 5–23% of women [12], with prevalence exceeding 50% in some countries [12,13,14]. In Russia, population-based studies of SDB have shown that snoring occurs in 58.2% of men and 51.8% of women, while OSA is present in 9.7% of men and 8.3% of women [15].

Position-dependent forms of sleep apnea occur in a significant proportion of patients with OSA. Epidemiological studies show that in approximately 53–75% of people with OSA, disease severity is dependent on body position, significantly increasing when sleeping in the supine position [16]. For such patients, maintaining a non-supine sleeping position may be the most effective treatment method, leading to a substantial reduction or complete elimination of adverse respiratory events. This therapeutic approach can be used as a standalone treatment or in combination with continuous positive airway pressure (CPAP) therapy. Unfortunately, CPAP, the treatment of choice for patients with moderate to severe OSA, shows low long-term adherence in this patient group [17,18,19]. This limitation has fueled growing interest in alternative treatments, including positional therapy (PT), which has emerged as a promising solution for position-dependent SDB. Notably, independent studies conducted by Dutch [20] and Danish [21] research groups have demonstrated significant clinical benefits of PT, including reduced time spent in a supine position, decreased apnea–hypopnea index values (a measure of disease severity), reduced excessive daytime sleepiness and improved overall daytime functioning.

Despite its potential, PT has not yet been widely accepted or implemented, either as a standalone or adjunctive treatment for POSA, including in Russia. Furthermore, the long-term efficacy of PT devices, as well as their overall impact on sleep architecture in patients with OSA, remains a topic of ongoing research.

The goal of this pilot study was to evaluate the efficacy of the SoftSleep vibrotactile device [SoftSleep v. 1.2.1, SoftSleep LLC, Moscow, Russia] in reducing time spent in the supine position without negatively impacting sleep quality in healthy volunteers. The specific objectives of the study were to quantify changes in the duration and proportion of sleep in the supine position using actigraphy monitoring, assess potential changes in total sleep time, and evaluate subjective sleep quality and tolerance to vibrotactile stimulation. In this context, the study was designed to evaluate sleep posture changes as a prerequisite for subsequent respiratory outcome assessment in patients with POSA. The results of this study may serve as a basis for subsequent clinical trials involving patients with POSA and contribute to the development of individualized non-pharmacological intervention strategies for patients with position-dependent SDB.

## 2. Results

Objective and subjective sleep parameters were analyzed in 20 volunteers across two conditions: a control night without the SoftSleep device and an experimental night with the SoftSleep PT device. All 20 volunteers from the original cohort completed the study and all three stages of the protocol.

### 2.1. Total Sleep Duration

**Figure 1:**
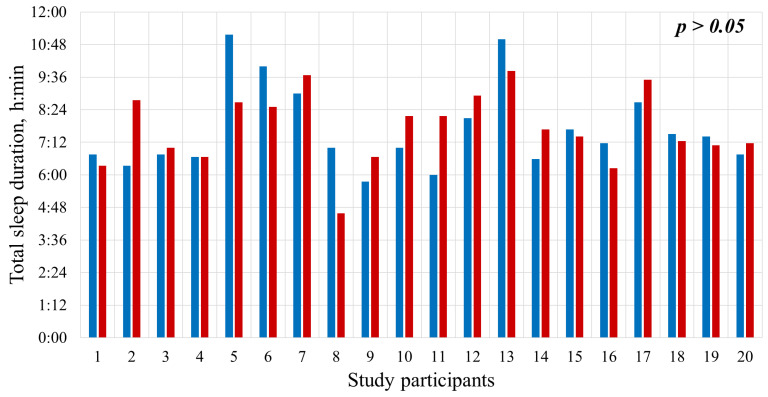
Total sleep time on the first night (blue bars) and the second night (red bars). h = hours; min = minutes.

Figure 1 illustrates the distribution of total sleep time for all study participants across two stages: total sleep time on the first night without the vibrotactile device (blue bars) and on the second night with the device (red bars). The mean total sleep time on the first night was 7:39 ± 1:33 h, and on the second night, 7:42 ± 1:19 h. As these data show, the average nightly sleep duration did not differ statistically significantly between the night without intervention and the night with PT, indicating that it was possible to make a reliable comparison of the time spent in the supine position and in the non-supine position for these study periods. This also indirectly indicates that the vibrotactile device did not affect total sleep duration. The differences between the two conditions did not reach statistical significance (*p* > 0.05), supporting the conclusion that the device did not alter sleep structure or architecture.

### 2.2. Duration of Sleep in the Supine Position

**Figure 2:**
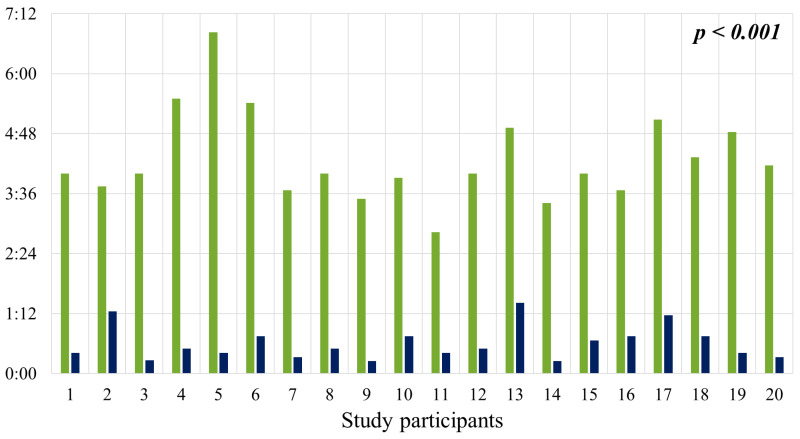
Duration of sleep in the supine position on the first night (green bars) and on the second night (dark blue bars). h = hours; min = minutes.

Figure 2 shows the amount of time participants spent sleeping in the supine position on the first night (green bars) and the second night (dark blue bars). On average, study subjects spent 4:17 ± 0:54 h in the supine position on the first night, compared to only 0:36 ± 0:20 h on the second night. This represents a statistically significant reduction in the amount of sleep time spent in the supine position with the SoftSleep device. On the control night, the average proportion of total sleep time spent in the supine position was 56.01%. The use of the SoftSleep device reduced this proportion to 7.84% (*p* < 0.001). This statistically significant change indicates a noteworthy shift in sleep patterns and high sensitivity of the study participants to the vibrotactile stimulus: the average reduction in the proportion of sleep spent in the supine position was 48.17 percentage points.

### 2.3. Total Number of Body Position Changes During Sleep

**Figure 3:**
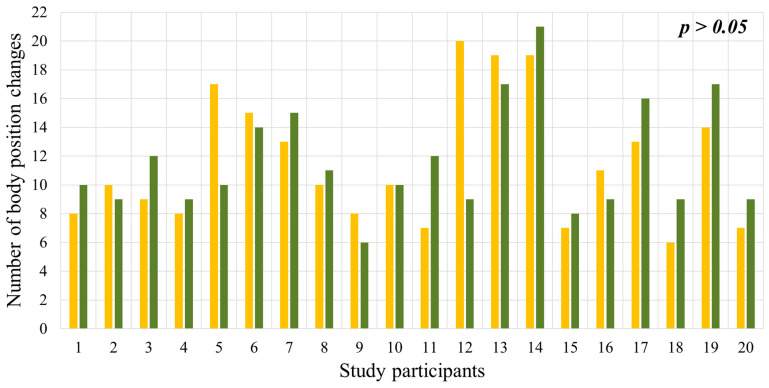
Number of body position changes on the first night (yellow bars) and on the second night (green bars). h = hours; min = minutes.

On the second night, we observed a moderate increase in body position changes, primarily in response to vibrotactile stimulation (Figure 3). Participants reported that these episodes were not accompanied by full awakenings and, in most cases, were not perceived as uncomfortable. These findings are consistent with the concept of brain microarousal described in the context of PT, in which stimulation induces short-term changes in motor activity without disrupting the overall sleep structure associated with awakening.

### 2.4. Assessment of Subjective Sleep Quality

**Figure 4:**
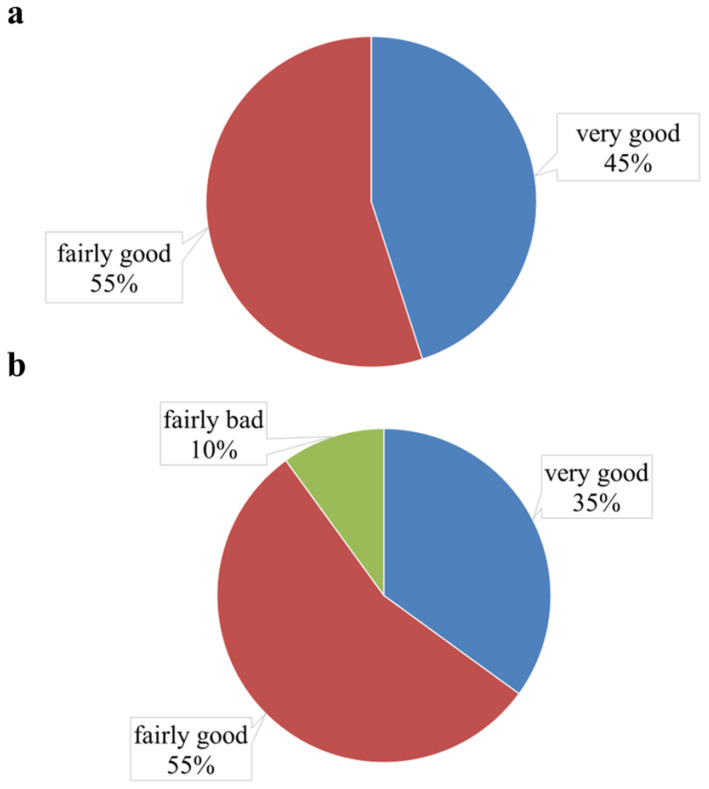
Sleep quality assessment using the modified Pittsburgh Sleep Quality Index: night without positional therapy (**a**) and night with a positional therapy device (**b**).

According to the Pittsburgh Sleep Quality Index, no significant differences were observed between the two conditions (Figure 4). On the control night, all participants rated their sleep as *very good* or *fairly good*, while on the second night, only 90% of them did: two participants noted a slight reduction in sleep quality on the second night, which they attributed to the need to adapt to wearing the device rather than to the actual vibrotactile effect.

### 2.5. The Presence of Nocturnal Awakenings Subjectively Associated with the Use of the Device

Three volunteers reported isolated episodes of awakening subjectively related to vibration. However, in none of these cases did the awakenings interrupt sleep for more than 2–3 min. All participants continued using the device for the rest of the night, and 90% reported no significant discomfort.

**Figure 5:**
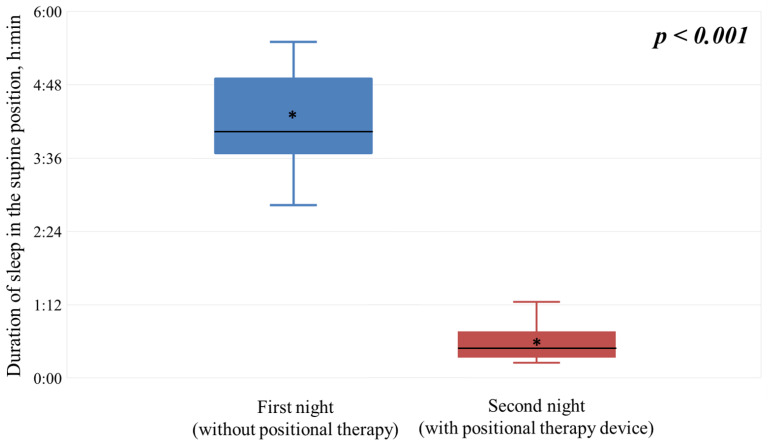
Summary data of sleep duration analysis in the supine position. *—mean value; h = hours; min = minutes.

Summary data from the time course analysis for the first two stages are presented in Figure 5.

## 3. Discussion

The objective of this pilot study was to evaluate whether a vibrotactile PT device could effectively reduce time spent in the supine position without negatively impacting sleep quality in healthy volunteers. Our findings demonstrate that the SoftSleep vibrotactile device can induce significant postural changes during sleep while maintaining total sleep duration and subjective sleep quality. In a two-day observational study combining actigraphy monitoring with subjective sleep quality assessment, our results supported the hypothesis that vibrotactile stimulation can induce significant body position changes during sleep (in the non-supine position) without negatively affecting subjective sleep perception or total sleep duration.

The device operates using a non-pharmacological intervention principle (vibrotactile feedback) that triggers brief activation of the central nervous system and a reflexive change in body position without awakening the sleeper. This is consistent with the microarousal theory described in several sleep medicine studies, which have shown that such microarousals do not disrupt sleep architecture, provided they are infrequent (less than 10 episodes per hour) and do not reach the level of full wakefulness [20,22].

The mean percentage of sleep time spent in the supine position decreased from 56.01% to 7.84%, suggesting an absolute reduction of 48.17 percentage points. These results are consistent with the results of international studies in which similar devices (e.g., Night Shift Sleep Positioner, Philips NightBalance) demonstrated a 10–15% reduction in sleep time in the supine position when using positional stimulation in patients with POSA [20,23]. Thus, the SoftSleep device exhibits equivalent efficacy while remaining a more affordable therapeutic option.

From a translational medicine perspective, POSA is characterized by a pronounced dependence of respiratory events on body position, particularly the supine position. Therefore, the device’s ability to reliably reduce time spent in the supine position represents a necessary behavioral prerequisite for subsequent improvement of respiratory parameters in POSA patients. In this context, the results obtained in healthy individuals support the concept of the behavioral effectiveness of vibrotactile PT and warrant further evaluation in clinical populations.

Of particular significance is the fact that total sleep time with the device did not differ from the control conditions (7:42 ± 1:19 vs. 7:39 ± 1:33), and no significant deterioration in sleep quality was detected according to the sleep quality questionnaire. In fact, the vast majority of participants (90%) rated their sleep with the device as *very good* or *fairly good*, thereby indicating the high tolerability and adaptability of the technology. These results confirm the potential of SoftSleep as a noninvasive therapy for patients with POSA.

It is also worth noting the variability in individual responses to the therapy: in some volunteers, the time spent sleeping in the supine position decreased to 1–2%, while in others it remained at 10–15%. This individual sensitivity may be influenced by differences in sleep depth (predominance of delta sleep or deeper slow-wave sleep stages), neurophysiological characteristics of tactile signal processing, or habitual baseline sleep position. A comprehensive assessment of these factors will require extensive clinical studies using polysomnography and objective assessment of sleep architecture.

It should also be noted that only three participants experienced brief vibration-related awakenings, none of which were perceived as uncomfortable and did not result in discontinuation of participation. This finding confirms the appropriateness of the selected stimulation zone (the lower sternum, where the device contacts the bony surface) and the adequacy of the vibration intensity settings.

Some publications have repeatedly emphasized that one of the disadvantages of most alternative CPAP therapy methods is low patient compliance with treatment [18,19]. In this short-term study, all participants used the SoftSleep device on both nights, indicating its good initial acceptability and tolerability. However, long-term compliance requires further study, particularly in patients with SDB.

Limitations of the current study include its small sample size (*n* = 20), reflecting the pilot nature of the study, the lack of sleep architecture analysis, and the selective assessment of sleep quality and wakefulness using the Pittsburgh Sleep Quality Index. It is important to note that the study was conducted in healthy volunteers. Although the observed behavioral effects demonstrate the feasibility and sensitivity of the device, its clinical efficacy in terms of respiratory outcomes in POSA patients is to be established in future patient studies. Furthermore, the short duration of the current study precludes an assessment of habituation to vibrotactile stimulation and the sustained effectiveness of PT. It is possible that behavioral responses to vibration may change over time due to sensory adaptation or learned avoidance of the supine position. Therefore, longitudinal studies with long-term monitoring periods are needed to assess the sustainability of the behavioral effect and the influence of habituation on therapeutic outcomes. We propose that the obtained results can serve as a basis for a subsequent pilot clinical trial in the target group of patients.

Thus, this study confirms that the SoftSleep vibrotactile device effectively reduces the duration of sleep in the supine position in volunteers without affecting total sleep time or subjective sleep quality. Its high tolerability and absence of side effects make it a promising tool for PT used for certain forms of snoring and OSA that are dependent on body position. Future studies should utilize a larger sample size, include clinical patient groups (particularly those with position-dependent SDB) and conduct a comprehensive polysomnographic evaluation to confirm these results and expand the indications for the use of next-generation vibrotactile devices in the treatment of POSA.

## 4. Materials and Methods

### 4.1. Study Design and Equipment

**Figure 6:**
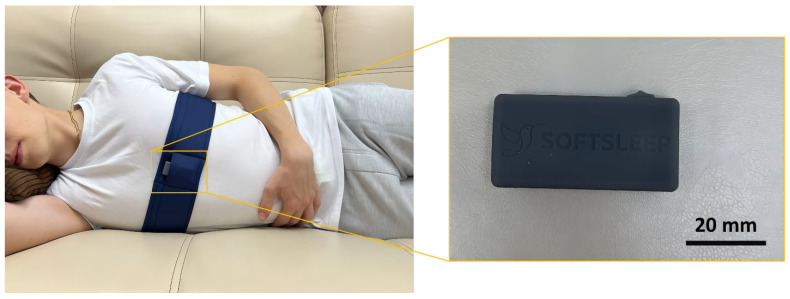
Appearance of the wearable vibrotactile device (SoftSleep) for preventing sleep-disordered breathing.

The pilot study was designed as a single-center, cross-sectional, clinical experimental study in a group of healthy volunteers. The study protocol consisted of three sequential stages, each designed to collect objective data on the participants’ body position during sleep, evaluate the device’s effects (Figure 6), and obtain subjective sleep quality assessments. The total study duration for each participant was two consecutive nights.

During the first night, participants underwent sleep monitoring using the Astrocard-SomnoSTUDIO automated polysomnography system [v. 3.1.18, Meditek LLC, Moscow, Russia], designed for outpatient assessment of SDB. The system is capable of actigraphy, i.e., recording blood oxygen saturation, respiratory airflow, thoracic respiratory effort, and body position during sleep). In this study, the device was used exclusively as a standard actimetry sensor, allowing for the collection of quantitative data on the duration of sleep in various body positions (supine, lateral recumbent, and prone) and the frequency of position changes throughout the night. Data collection was continuous throughout the night under conditions as close as possible to the participants’ natural sleep environment.

The second night involved the simultaneous use of two devices: the Astrocard-SomnoSTUDIO system and the SoftSleep PT device. The SoftSleep device [SoftSleep v. 1.2.1, SoftSleep LLC, Moscow, Russia] is a vibrotactile module secured to the chest with an elastic textile strap. Its primary function is to prevent patients from sleeping in a supine position while maintaining clinical effectiveness. Throughout the night, the SoftSleep device continuously monitors body position, classifying it into one of four categories: supine, right lateral, left lateral, or prone. Upon detecting a supine position, the device’s control module initiates vibration stimulation. The SoftSleep device is compact (44 × 22 × 11 mm), weighs approximately 21 g, and is powered by a rechargeable lithium-polymer battery providing up to 12 h of continuous operation. The data transmission module delivers a wireless connection to a mobile app, allowing both data transfer to a smartphone and customization of the vibrotactile device’s parameters (e.g., vibration intensity, startup delay time) to meet the user’s individual needs.

To ensure proper placement and minimize measurement artifacts, all devices were attached to the study participant after preliminary briefing and under the supervision of the researcher. The briefing included instructions for using the equipment, safety precautions for operating the vibrotactile device, and recommendations for handling the medical system for recording sleep parameters. The SoftSleep device was positioned in the area of the xiphoid process, and the Astrocard-SomnoSTUDIO system was placed above the SoftSleep belt so as not to interfere with its operation.

The third stage, conducted in the morning after each night of the study, involved completing a modified version of the Pittsburgh Sleep Quality Index (Section 6) [24]. This assessment included a subjective evaluation of sleep quality on a four-point scale: *very good*, *fairly good*, *fairly poor*, and *very poor*. In addition, participants were asked to indicate the presence or absence of awakenings associated with vibration stimuli and to rate their overall satisfaction with the quality of their rest at night. It is worth noting that the overall Pittsburgh Sleep Quality Index score was not calculated because the questionnaire is validated to assess sleep quality over a two-week period, rather than over a two-day study (in our case).

The devices were returned to the researchers two nights later to transfer the data, analyze the recordings, and check the accuracy of the overlay.

### 4.2. Recruitment and Monitoring of Volunteers

Participants were selected using a non-probability convenience sampling method. The study included 20 volunteers who did not report active health complaints at the time of inclusion in the study. Participants were recruited based on voluntary participation following an informational event, and no randomization procedure was employed.

Before inclusion in the study, each participant underwent an initial interview, medical history collection, and subjective health assessment. Inclusion criteria were as follows: (1) age ≥ 18 years; (2) self-assessed good general health; (3) ability to comply with the study protocol; and (4) provision of written informed consent. Exclusion criteria encompassed: (1) known chronic somatic or psychiatric disorders; (2) suspected OSA based on a screening questionnaire; (3) previously diagnosed sleep disorders; and (4) use of medications affecting sleep architecture. Participants who reported snoring but had no other clinical signs of SDB were eligible for inclusion in the study.

Our research was designed as a pilot exploratory study. No formal sample size calculation was performed, as the primary objective was to evaluate the feasibility and identify a significant within-subject effect of vibrotactile PT on sleep duration in the supine position.

The study was approved by the Ethics Committee of the National Medical Research Center for Therapy and Preventive Medicine (Protocol No. 02-03/22, 17 March 2022, Moscow, Russia), and all experimental procedures were conducted in accordance with the ethical standards outlined in the Declaration of Helsinki. Prior to the study, all volunteers were informed of its objectives, methodology, and potential risks. Each participant provided written informed consent in accordance with the principles of the Declaration of Helsinki.

The study cohort included 14 men (70%) and 6 women (30%). Six participants were aged 18–34 years (young adult age group), 12 were aged 35–59 years (middle-age group), and two were over 60 years (older adults). The mean age of the participants was 41.8 ± 11.2 years. According to the questionnaire, eight participants (40%) reported occasional snoring, which was confirmed by their bed partners. A summary of the participant characteristics is presented in Table 1.

Sleep parameter recordings using the Astrocard-SomnoSTUDIO diagnostic system and the SoftSleep therapeutic device were performed at the participants’ homes without any restrictions on physical activity or changes to their usual sleep patterns. No pharmaceuticals potentially affecting sleep structure (including hypnotics, sedatives, anxiolytics, etc.) were prescribed or used at least 72 h before the start of the study and during the entire study period.

### 4.3. Outcome Measures

The efficacy of the SoftSleep vibrotactile device was assessed using a combination of objective and subjective parameters characterizing the participant’s sleep position and overall sleep quality. In this pilot study, we did not assess key physiological parameters during sleep, including breathing patterns. The primary outcome measures were as follows:

1. *Total sleep duration* (hours and minutes). This parameter was calculated based on actigraphy data obtained with the Astrocard-SomnoSTUDIO system;
2. *Sleep duration in the supine position* (in minutes and as a percentage of total sleep time). This parameter was calculated based on actigraphy data recorded by the Astrocard-SomnoSTUDIO system. Comparisons were made between two nights: without PT (control value) and with PT;
3. *Total number of body position changes during sleep*. This parameter served as an indicator of the participant’s motor activity. An increase in the number of body position changes, accompanied by a reduction in sleep time in the supine position, was interpreted as the targeted effect (provided it was not associated with a noteworthy deterioration in subjective sleep quality);
4. *Subjective sleep quality assessment based on Section 6 of the Pittsburgh Sleep Quality Index.* Volunteers completed a questionnaire after each night, rating their overall sleep experience on a four-point scale. Particular attention was paid to instances in which sleep ratings worsened during the experimental night;
5. *Presence and frequency of nocturnal awakenings subjectively related to device operation*. This parameter was assessed using an additional questionnaire, which included questions about the occurrence, number, and duration of vibration-induced awakenings. Awakenings resulting in a deterioration in overall sleep quality were recorded separately.

The efficacy of the SoftSleep device was defined as a statistically significant reduction in sleep time in the supine position without a corresponding deterioration in subjective sleep quality. This approach is consistent with criteria adopted in previous studies evaluating PT devices [20,21].

### 4.4. Statistical Analysis

Data processing and analysis were performed using Microsoft Excel 2019 [Microsoft Corporation, Washington, DC, USA] and MathWorks MATLAB R2023a [MathWorks, Natick, MA, USA]. All quantitative variables were checked for outliers and assessed for normality using visual analysis of histograms and Q-Q plots.

Sample characteristics are presented as mean values and standard deviations (M ± SD, where M is the mean and SD is the standard deviation) for quantitative variables. To compare two conditions (nights with and without the SoftSleep device), a paired Student’s *t*-test for dependent samples was used. In cases of substantial deviations from normality, an alternative nonparametric analysis was performed using the Wilcoxon signed-rank test. Taking into account the pilot nature of the study and the limited sample size, a statistical significance threshold of *p* < 0.001 was utilized to reduce the likelihood of false-positive test results for multiple related outcome measures.

Data visualization was performed using the built-in analysis tools of the MATLAB R2024b software package. The results included comparative graphs for the main parameters: total sleep duration, sleep duration in the supine position, and the number of body position changes. The obtained data were then compared with the results of similar studies on the efficacy of vibrotactile PT devices published in international publications. Qualitative variables (e.g., subjective sleep quality ratings) were expressed as percentages across conditions and were presented as pie and bar charts.

## 5. Conclusions

The results of this study demonstrated the high efficacy and good tolerability of the SoftSleep vibrotactile device developed for positional therapy of SDB and snoring prevention. Use of the device significantly reduced the duration of supine sleep without negatively impacting total sleep time or sleep quality.

A more than sevenfold mean reduction (from 56% to 7.8%) in the proportion of sleep duration in the supine position confirms the ability of SoftSleep to effectively develop a stable behavioral pattern of avoiding sleep in an unfavorable (non-supine) position. The absence of significant side effects and high participant compliance highlight the potential of this device as a non-pharmacological intervention that provides a high degree of comfort and safety.

Our findings support the feasibility of further clinical trials involving patients diagnosed with OSA. In the future, the SoftSleep device may find a place in the treatment armamentarium for mild to moderate positional forms of OSA, particularly in patients with poor compliance to CPAP therapy.

## Figures and Tables

**Table 1:** Characteristics of the study participants.

Total Number of Volunteers	20
Male/Female	14/6
Age groups (age range, years):	
18–34	6
35–59	12
60 and older	2
Mean body mass index (BMI), kg/m^2^ ± SD	24.8 ± 2.9
Participants reporting snoring	8 (40%)

## Data Availability

The data supporting the results of this study are available upon reasonable request from the corresponding author.
